# Biochemical predictors of structural hypothalamus–pituitary abnormalities detected by magnetic resonance imaging in men with secondary hypogonadism

**DOI:** 10.1007/s40618-021-01586-5

**Published:** 2021-05-10

**Authors:** S. Cipriani, T. Todisco, N. Ghiandai, L. Vignozzi, G. Corona, M. Maggi, G. Rastrelli

**Affiliations:** 1 Andrology, Women’s Endocrinology and Gender Incongruence Unit, Department of Experimental Clinical and Biomedical Sciences “Mario Serio”, Careggi Hospital, University of Florence, Florence, Italy; 2 I.N.B.B., Istituto Nazionale Biostrutture e Biosistemi, Rome, Italy; 3 Endocrinology Unit, Medical Department, Azienda Usl Bologna Maggiore-Bellaria Hospital, Bologna, Italy; 4 Endocrinology Unit, Department of Experimental Clinical and Biomedical Sciences “Mario Serio”, Careggi Hospital, University of Florence, Florence, Italy

**Keywords:** Male secondary hypogonadism, Hypothalamus–pituitary abnormalities, MRI of hypothalamic–pituitary region, Pathological findings on pituitary MRI, Biochemical predictors, Hormonal thresholds

## Abstract

**Purpose:**

Organic conditions underlying secondary hypogonadism (SH) may be ascertained by magnetic resonance imaging (MRI) of the hypothalamic–pituitary region that could not be systematically proposed to each patient. Based upon limited evidence, the Endocrine Society (ES) guidelines suggest total testosterone (T) < 5.2 nmol/L to identify patients eligible for MRI. The study aims to identify markers and their best threshold value predicting pathological MRI findings in men with SH.

**Methods:**

A consecutive series of 609 men seeking medical care for sexual dysfunction and with SH (total T < 10.5 nmol/L and LH ≤ 9.4 U/L) was retrospectively evaluated. An independent cohort of 50 men with SH was used as validation sample. 126 men in the exploratory sample and the whole validation sample underwent MRI.

**Results:**

In the exploratory sample, patients with pathological MRI findings (*n* = 46) had significantly lower total T, luteinizing hormone (LH), follicle stimulating hormone (FSH) and prostate specific antigen (PSA) than men with normal MRI (*n* = 80). Receiver Operating Characteristics analysis showed that total T, LH, FSH and PSA are accurate in identifying men with pathologic MRI (accuracy: 0.62–0.68, all *p* < 0.05). The Youden index was used to detect the value with the best performance, corresponding to total T 6.1 nmol/L, LH 1.9 U/L, FSH 4.2 U/L and PSA 0.58 ng/mL. In the validation cohort, only total T ≤ 6.1 nmol/L and LH ≤ 1.9 U/L were confirmed as significant predictors of pathologic MRI.

**Conclusion:**

In men with SH, total T ≤ 6.1 nmol/L or LH ≤ 1.9 U/L should arise the suspect of hypothalamus/pituitary structural abnormalities, deserving MRI evaluation.

## Introduction

Low testosterone (T) with inappropriately normal or low gonadotropins (secondary hypogonadism) is a frequent finding with a prevalence of 11% in general population [1] and 17% among patients consulting for sexual dysfunction [2]. It may derive from structural abnormalities in the hypothalamus or pituitary, including potentially progressive and health threatening lesions, such as macroadenomas or craniopharyngiomas, or benign conditions that, however, may negatively affect over time other hypothalamus–pituitary axes, such as empty sella, head trauma or radiotherapy. More often [3], secondary hypogonadism develops in association with comorbidities, including obesity or diabetes mellitus as a result of the underlying pathogenic mechanism (e.g., insulin resistance, chronic systemic inflammation, use of medications affecting the hypothalamic–pituitary–testicular axis [HPT]) in men with otherwise normal hypothalamus and pituitary. The former condition is commonly referred to as organic hypogonadism, whereas the latter is defined as functional hypogonadism [4]. Differentiating organic from functional forms is paramount, because there is growing opinion that functional hypogonadism should be better managed through the treatment of the underlying chronic disease (i.e., lifestyle measures, optimization of medical treatment, etc.) rather than by T replacement therapy (TRT) [4]. In fact, weight loss or measures leading to the improvement in comorbidities are able to remove the inhibitory signals on the HPT axis, which lead to secondary hypogonadism [5].

The definitive classification of hypogonadism as functional is a diagnosis of exclusion. In fact, having a condition known to affect negatively the HPT is not sufficient to exclude anatomical abnormalities. Consequently, a hypothalamus–pituitary magnetic resonance imaging (MRI) should be required in any secondary hypogonadal men. Indeed, this is only ideally feasible, because MRI is an expensive procedure, not widespread available and still burdened by long waiting lists so that this should be reserved to selected secondary hypogonadal patients with a greater a priori probability of hypothalamic–pituitary abnormalities. The attempt to outline the features that differentiate functional from organic hypogonadism led to recognize empirically that the former is commonly characterized by higher T levels fluctuating around the lower limit of normality with relatively normal gonadotropins [4, 6]. Conversely, organic hypogonadism may be suspected for unequivocally and severely low T with inappropriately normal or low gonadotropins [4, 6]. The Endocrine Society (ES) guidelines [7] suggest, in absence of neurological disturbances, 5.2 nmol/L as the value of total T below which an organic structural alteration in the hypothalamus–pituitary may be suspected and that, therefore, may deserve MRI testing. The identification of this threshold of total T derives from a single study [8] performed on a limited series of men from a urological service that may be not representative of patients evaluated in an endocrine practice. More importantly, the value identified did not find consistent confirmation in following studies, and therefore, it has not been sufficiently validated for the use in clinical practice. According to the poor evidence upon which the threshold 5.2 nmol/L of total T is based, an online survey conducted on endocrinologists and andrologists affiliated to the major international professional societies showed that only 20% of the responders use total T < 5.2 nmol/L for requiring MRI to secondary hypogonadal men, whereas almost 45% adopt higher T values [9].

This study aimed to evaluate formally the performance of total T 5.2 nmol/L in a population of men with secondary hypogonadism and consistent sexual symptoms. In addition, the study aimed to identify possible markers and their best threshold values that predict anatomical hypothalamic–pituitary abnormalities thus possibly helping in addressing the clinical decision of performing an MRI in men with secondary hypogonadism. The identification of these values were conducted in an exploratory sample and afterwards validated in an independent validation cohort to corroborate the results.

## Materials and methods

### UNIFI cohort

A consecutive series of 609 men seeking medical care for sexual dysfunction at the Andrology Unit of the Careggi University Hospital (University of Florence, Italy) and classified as secondary hypogonadal for total T < 10.5 nmol/L and luteinizing hormone (LH) ≤ 9.4 U/L, according to the European Male Ageing Study (EMAS) [1], were analyzed because of suspected functional or organic causes of secondary hypogonadism. None of these had neurological symptoms, headache or visual defects. At the first visit, before starting any treatment for sexual dysfunction, all patients underwent a standard diagnostic protocol including validated structured interviews for the characterization of erectile disfunction (ED) [10] and for the assessment of clinical features consistent with androgen deficiency [11]. In addition, medical history, including comorbidities or medications taken, and a complete physical examination were performed. Metabolic and hormone parameters were measured on fasting blood samples drawn in the morning before 10 a.m. All measurements were performed at the central laboratory of our University Hospital, which participates routinely in external and internal quality control programs. Total T, sex hormone binding globulin (SHBG), LH and follicle stimulating hormone (FSH) were measured by an immunoassay (Modular E170 platform electrochemiluminescence immunoassays-Roche Diagnostics-Mannheim, Germany). The intra and inter-assay coefficients of variation for total T are 1.05% and 3.72% at 14.4 nmol/L. Free T was calculated according to the Vermeulen’s formula [12]].

A possible organic cause of secondary hypogonadism was investigated by MRI of the hypothalamus–pituitary region according to the judgement and personal experience of the physician. The team working at our center and managing these specific patients includes five endocrinologists, all with similar professional seniority and scientific background. All MRIs requested were performed with contrast media by qualified radiologic institutes or hospital radiology divisions in Florence or in the surrounding area. Of the 126 collected MRIs in eligible patients (exploratory sample), 46 (36.5%) reported pathologic findings. In particular, 15 reported microadenomas, six macroadenomas, 16 empty sella (13 idiopathic and three post-surgery), two pituitary hypoplasia, two Rathke’s cleft cysts, four pituitary stalk diseases (including one post-traumatic lesion, one thickening and two disruption without evidence of pituitary masses), and one described radiology signs of iron overload in a patient with history of chronic blood transfusion therapy.

### Bologna Maggiore Hospital cohort

An independent cohort of 50 men consulting for sexual dysfunction at the Endocrinology Unit of Maggiore Hospital (Bologna, Italy) and with ascertained secondary hypogonadism required to perform an MRI for ruling out organic conditions affecting the hypothalamus and pituitary were used as a validation cohort. In the center, patients were evaluated by a single endocrinologist. Patients were assessed with a similar diagnostic protocol as in Florence, including structured interviews, medical history, physical examination and serum parameters, as part of routine clinical evaluations. In particular, as in the UNIFI cohort, information from men with total T < 10.5 nmol/L and LH ≤ 9.4 U/L required with MRI were retrospectively and consecutively collected. All MRIs requested were performed with contrast media by qualified radiologic institutes or hospital radiology divisions in Bologna or the surrounding area. In the validation sample, the following pathologic findings were reported at MRI: 10 microadenomas, 5 macroadenomas, 7 empty sella (6 idiopathic and 1 post-surgery outcome), 1 Rathke’s cleft cyst, 3 pituitary hypoplasia, 2 pituitary stalk diseases.

### Statistical methods

Data were reported as means ± standard deviation when normally distributed, median [interquartile range] when non-normally distributed, and percentage when categorical. The differences between groups have been evaluated by *χ*^2^ test for categorical variables and by *t* test for continuous ones. For non-normally distributed parameters, log-transformation of the values was performed to achieve normality.

Non-linearity of the relationships among serum parameters and the probability of pathological MRI findings was evaluated by the locally weighted scatterplot smoothing (LOWESS). Afterwards, threshold levels for serum markers were identified on the LOWESS and further tested by regression models with linear-spline functions for the independent variables. This analysis allowed identifying threshold levels at which a significant change in the slope of the association between the independent variable and the probability of pathologic MRI occurred.

Receiver Operating Characteristic (ROC) analysis has been used for evaluating the accuracy (expressed as area under the curve; AUC) of relevant serum parameters in discriminating men with pathologic findings at hypothalamus–pituitary MRI. The coordinates of the ROC curve have been used for calculating the Youden index and identifying the threshold values with the best performance.

Through the LOWESS and ROC analysis, a series of possible threshold values were identified. The choice on the most proper one was taken posing more value on the operating characteristics. The thresholds thus identified in the exploratory sample were tested in the validation sample by logistic regression models, evaluating the odds ratio (OR) and 95% confidence interval (CI) of having a pathologic finding at MRI.

Hyperprolactinemia may be a misleading biochemical sign due to the non-negligible occurrence of non-pathologic high prolactin (i.e., macroprolactinemia, venipuncture) [13]. For this reason, to provide useful thresholds independently of prolactin levels and thus possibly offering additive information to prolactin, we decided not to exclude hyperprolactinemic men from the main analysis. However, the thresholds obtained in the exploratory cohort and their performance in the validation cohort were checked in a sensitivity analysis, excluding men with overt hyperprolactinemia (prolactin ≥ 735 mU/L).

All statistical analyses were conducted using Stata MP 13.1 for Windows (StataCorp, College Station, TX, USA) and *p* values < 0.05 were considered statistically significant. All figures were produced using GraphPad Prism 5.02.

## Results

### UNIFI cohort

In the UNIFI cohort, 154 MRI (25.3%) were requested. Table 1 shows the differences between men who were requested to perform an MRI of the hypothalamus–pituitary and those for whom MRI was considered unnecessary by the physician. Men required to perform an MRI were younger, had a slightly—although statistically significant—higher testis volume, lower LH and FSH as well as lower total and free T (Table 1). Accordingly, they had significantly lower prostatic specific antigen (PSA) and higher prevalence of hypoactive sexual desire, at variance with severe ED and impaired morning erections that were less frequent among men required to perform MRI (Table 1). Despite prolactin levels were similar in both groups, as well as the prevalence of subjects who took medications affecting prolactin secretion, the prevalence of severe hyperprolactinemia was significantly higher in men requested to perform a MRI (Table 1). Concerning metabolic parameters, men requested to perform MRI had lower total cholesterol and glucose, as well as a lower prevalence of diabetes mellitus (Table 1).

**Table 1:** Characteristics of patients with secondary hypogonadism from the UNIFI cohort who were or not required to undergo hypothalamic–pituitary MRI according to clinical practice

	MRI not requested (*n* = 455)	MRI requested (*n* = 154)	*p*
Age (years)	54.6 ± 11.4	50.5 ± 11.2	** < 0.0001**
Current smoker (%)	23.2	22.7	0.909
Alcohol intake (> 4 drink/day) (%)	3.6	3.9	0.824
Testis volume (mL)	19.0 ± 4.5	20.0 ± 4.4	**0.022**
Waist circumference (cm)	103.3 ± 12.6	102.1 ± 12.9	0.356
Waist circumference > 102 cm (%)	44.7	39.9	0.315
BMI (kg/m^2^)	28.8 ± 4.9	28.3 ± 5.2	0.382
Obesity (BMI > 30 kg/m^2^) (%)	32.0	29.4	0.559
Hypertension (%)	86.4	81.4	0.141
Luteinizing hormone (U/L)*	3.5 [2.4–5.0]	2.1 [1.4–3.0]	** < 0.0001**
Follicle stimulating hormone (U/L)*	4.7 [3.2–7.7]	3.6 [2.4–4.8]	** < 0.0001**
Thyroid stimulating hormone (mU/L)*	1.5 [1.1–2.1]	1.4 [0.9–2.1]	0.185
Adrenocorticotropic hormone (pg/mL)*	24.8 [16.0–34.6]	22.6 [16.0–31.0]	0.761
Cortisol (nmol/L)	404.6 ± 91.8	356.2 ± 126.8	0.326
Insulin-like Growth Factor-1 (ng/mL)	148.1 ± 62.2	185.4 ± 47.8	0.159
Prolactin (mU/L)*	145.0 [107.0–212.0]	156.0 [105.8–248.0]	0.168
Overt hyperprolactinemia (PRL > 735 mU/L) (%)	1.9	7.3	**0.001**
Antipsychotics (%)	3.5	3.2	0.852
Prokinetics (%)	0.0	2.4	**0.001**
SSRI (%)	7.5	7.1	0.900
Total testosterone (nmol/L)	8.5 ± 1.6	7.4 ± 2.0	** < 0.0001**
Sex hormone binding globulin (nmol/L)	26.4 ± 10.9	26.9 ± 18.4	0.759
Calculated free testosterone (pmol/L)	189.7 ± 43.2	167.5 ± 51.1	** < 0.0001**
Hemoglobin (g/dL)	15.0 ± 1.3	15.0 ± 1.0	0.934
Prostate specific antigen (ng/mL)*	0.8 [0.5–1.5]	0.7 [0.4–1.2]	**0.045**
Total cholesterol (mg/dL)	208.4 ± 45.6	199.9 ± 39.9	0.056
HDL-cholesterol (mg/dL)	46.0 ± 10.9	46.6 ± 11.8	0.599
Triglycerides (mg/dL) *	135.5 [100.0–194.8]	130.0 [102.0–184.5]	0.436
Fasting glucose (mg/dL)	110.8 ± 40.2	103.3 ± 30.6	**0.022**
Diabetes mellitus (%)	26.4	14.7	**0.006**
Severe erectile dysfunction (%)	75.5	66.0	**0.024**
Hypoactive sexual desire (%)	23.9	34.6	**0.010**
Impaired morning erection (%)	77.0	67.8	**0.024**
Perceived reduced ejaculate volume (%)	41.7	46.3	0.358

Differences between the two groups were evaluated by *χ*^2^ test (categorical variables) and by *t* test (continuous variables). Non-normally distributed parameters were log-transformed to achieve normality. *p* values are expressed in bold numbers when statistically significant

*MRI* magnetic resonance imaging, *BMI* body mass index, *PRL* prolactin, *SSRI* selective serotonin reuptake inhibitor, *HDL* high-density lipoprotein

*Non-normally distributed parameters

Among 154 men requested to perform a pituitary MRI, 126 (81.8%) followed the indication. The remaining 28 men that did not perform the required MRI differed only for younger age (46.8 ± 11.8 vs. 51.4 ± 10.9 years, *p* = 0.048). Of the 126 secondary hypogonadal men who represent the analytical sample, 46 (36.5%) had pathologic findings at MRI. The specific findings were reported in the “Materials and methods” section. Men with pathologic MRI findings had significantly lower LH, FSH, total T and PSA (Table 2). For this reason, these parameters were evaluated for their performance in predicting pathological MRI findings.

**Table 2:** Characteristics of patients in the UNIFI cohort who performed hypothalamic–pituitary MRI

	MRI normal (*n* = 80)	MRI pathological findings (*n* = 46)	*p*
Age (years)	52.1 ± 10.5	50.0 ± 11.6	0.306
Current smoker (%)	23.8	21.7	0.796
Alcohol intake (> 4 drink/day) (%)	6.3	0.0	0.160
Testis volume (mL)	20.2 ± 4.1	19.7 ± 4.7	0.492
Waist circumference (cm)	100.7 ± 11.7	103.7 ± 14.1	0.230
Waist circumference > 102 cm (%)	35.6	46.5	0.247
BMI (kg/m^2^)	27.7 ± 4.6	29.1 ± 5.7	0.161
Obesity (BMI > 30 kg/m^2^) (%)	28.8	34.9	0.492
Hypertension (%)	85.1	82.2	0.674
Luteinizing hormone (U/L)*	2.5 [1.5–3.4]	1.9 [1.3–2.8]	**0.007**
Follicle stimulating hormone (U/L)*	4.0 [2.8–5.0]	3.3 [2.0–4.0]	**0.002**
Thyroid stimulating hormone(mU/L)*	1.3 [0.9–2.2]	1.4 [0.9–2.0]	0.979
Adrenocorticotropic hormone (pg/mL)*	22.6 [20.6–58.0]	16.0 [13.7–30.4]	0.154
Cortisol (nmol/L)	308.0 ± 99.8	383.7 ± 151.8	0.366
Insulin-like Growth Factor-1 (ng/mL)	140.0 ± 30.8	203.5 ± 44.2	0.064
Prolactin (mU/L)*	168.0 [103.0–256.0]	157.4 [114.0–618.5]	0.348
Overt hyperprolactinemia (PRL > 735 mU/L) (%)	2.5	20.5	**0.001**
Antipsychotics (%)	5.0	0.0	0.123
Prokinetics (%)	2.5	2.2	0.699
SSRI (%)	6.3	8.7	0.608
Total testosterone (nmol/L)	7.7 ± 1.6	6.6 ± 2.5	**0.010**
Sex hormone binding globulin (nmol/L)	24.9 ± 10.7	25.4 ± 7.5	0.830
Calculated free testosterone (pmol/L)	172.9 ± 45.1	154.8 ± 56.1	0.124
Hemoglobin (g/dL)	14.9 ± 0.8	15.4 ± 0.9	0.213
Prostate specific antigen (ng/mL)*	0.8 [0.5–1.4]	0.5 [0.3–1.0]	**0.023**
Total cholesterol (mg/dL)	203.5 ± 36.1	198.8 ± 38.2	0.528
HDL-cholesterol (mg/dL)	48.8 ± 12.7	44.7 ± 11.0	0.105
Triglycerides (mg/dL)*	134.0 [102.0–187.3]	125.5 [95.0–185.0]	0.713
Fasting glucose (mg/dL)	101.7 ± 20.5	96.9 ± 17.7	0.220
Diabetes mellitus (%)	12.9	15.4	0.713
Severe erectile dysfunction (%)	67.5	61.9	0.537
Hypoactive sexual desire (%)	33.8	39.1	0.544
Impaired morning erection (%)	68.4	73.3	0.560
Perceived reduced ejaculate volume (%)	45.1	45.9	0.931

Differences between the two groups were evaluated by *χ*^2^ test (categorical variables) and by *t* test (continuous variables). Non-normally distributed parameters were log-transformed to achieve normality. *p* values are expressed in bold numbers when statistically significant

*MRI* magnetic resonance imaging, *BMI* body mass index, *PRL* prolactin, *SSRI* selective serotonin reuptake inhibitor, *HDL* high-density lipoprotein

*Non-normally distributed parameters

**Fig. 1:**
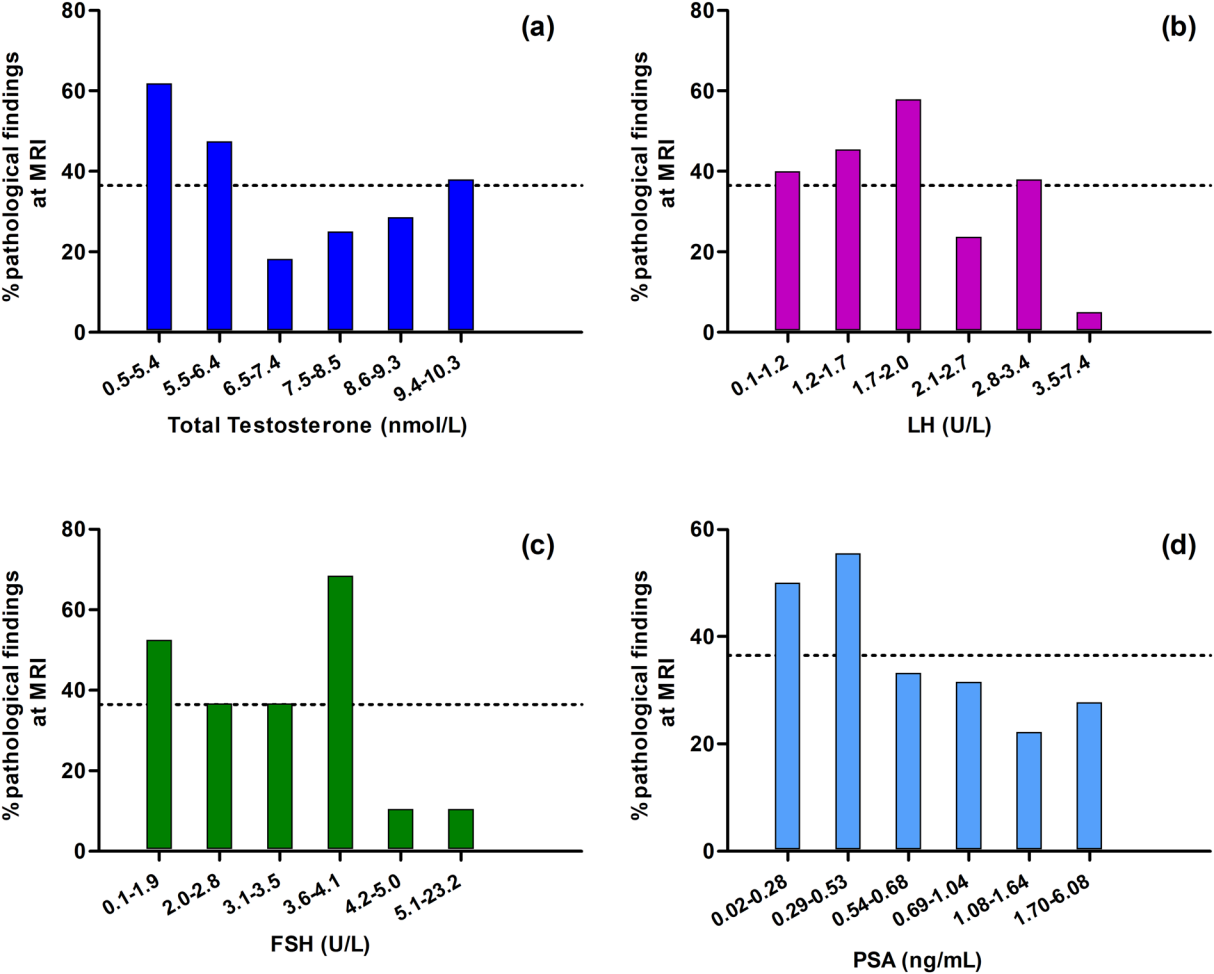
Observed prevalence of pathological findings at hypothalamic–pituitary MRI according to sextiles of total testosterone (**a**), LH (**b**), FSH (**c**) and PSA (**d**). The dotted line indicates the background prevalence of pathologic MRI in the UNIFI cohort. *MRI* magnetic resonance imaging, *LH* Luteinizing hormone, *FSH* Follicles stimulating hormone, *PSA* Prostatic specific antigen

Figure 1 shows the prevalence of pathologic hypothalamus–pituitary MRI findings according to sextiles of total T, LH, FSH and PSA. For total T, the background prevalence of pathologic MRI in the analytical population was consistently exceeded by the prevalence reported in the first two sextiles, whose upper limit corresponds to 6.4 nmol/L. For LH, FSH and PSA, the prevalence in the third, fourth and second sextiles, respectively, exceeded the background prevalence and their upper limits are LH = 2.0 U/L, FSH = 4.1 U/L and PSA = 0.53 ng/mL.

**Fig. 2:**
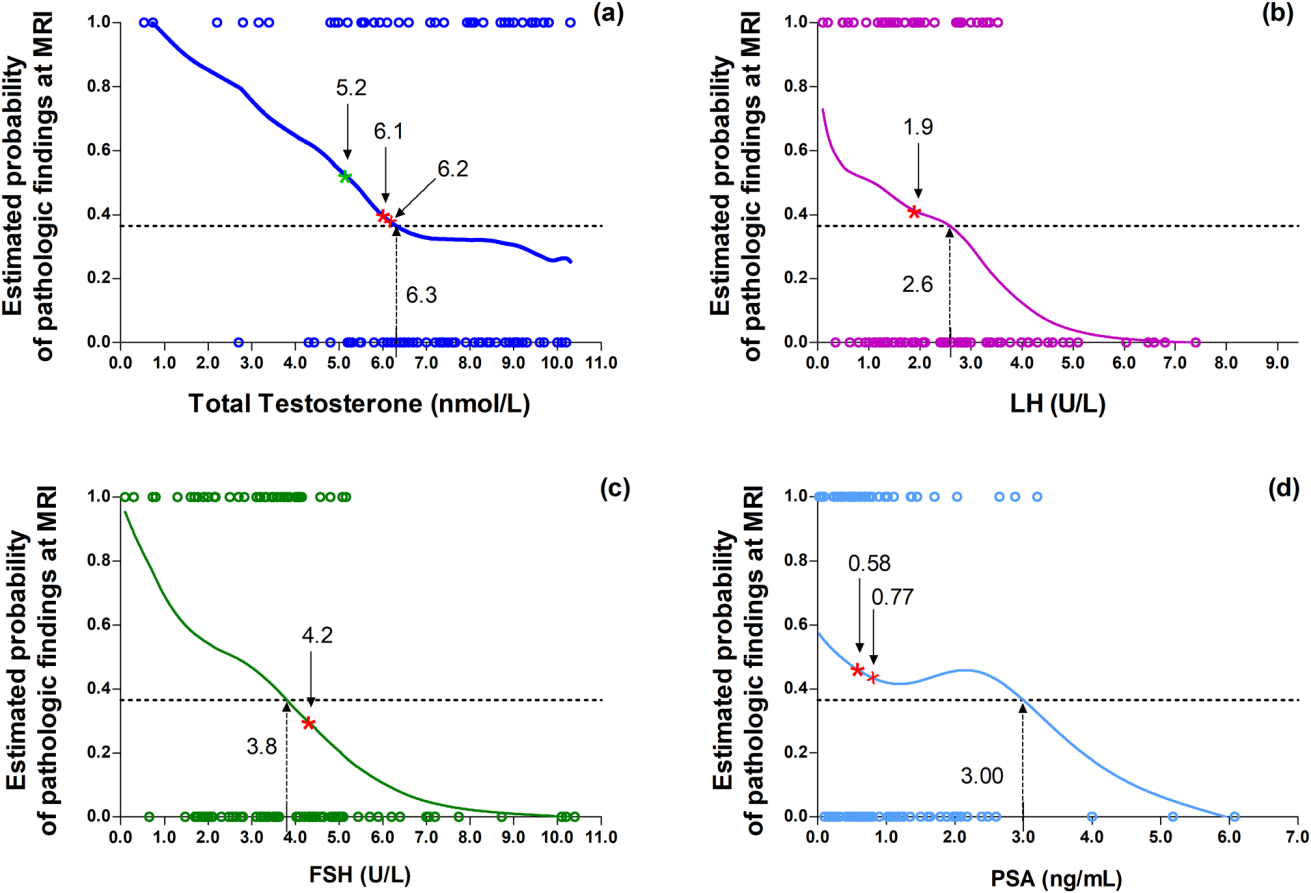
Relationship between total testosterone (**a**), LH (**b**), FSH (**c**), PSA (**d**) and the estimated probability of pathologic findings at hypothalamic–pituitary MRI. The smooth curves were carried out as locally weighted scatterplot smoothing (LOWESS). The dotted line indicates the background prevalence of pathologic MRI in the UNIFI cohort. The dotted arrow indicates the value corresponding to the background prevalence of pathologic MRI. The red cross denote the threshold value identified by modeling the regressions with linear spline functions (a threshold was identified for total testosterone and PSA but not LH and FSH). The red asterisk corresponds to the value with the best Youden index identified with the Receiver Operating Characteristics analysis (see also Fig. 3). The green asterisk in (**a**) denotes the threshold value of total testosterone suggested by the Endocrine Society [7]]. *MRI* magnetic resonance imaging, *LH* Luteinizing hormone, *FSH* Follicles stimulating hormone, *PSA* Prostatic specific antigen

For a more precise view of the possible threshold values in a continuous scale, we fitted LOWESS curves for the relationship between total T, LH, FSH and PSA with the estimated prevalence of a pathologic MRI. Figure 2a shows that the background prevalence of pathologic MRI is reached at total T = 6.3 nmol/L. In addition, it suggests the nonlinearity of the relationship between total T and the estimated probability of pathologic MRI. Linear regressions with linear spline function identified a threshold value at total T = 6.2 nmol/L: below this value, each 1 nmol/L decrease in total T was associated with a significant increase in the probability of pathological MRI findings (*B* = 0.14 [0.05;0.23], *p* = 0.002), whereas above 6.2 nmol/L the relationship was not significant (*B* = 0.00 [− 0.06;0.07]; *p* = 0.863). Furthermore, at total T = 6.2 nmol/L, the linear regression of the estimated probability of pathologic MRI based on linear spline functions showed a significant change in the slope of the relationship (*B* = 0.14 [0.00;0.27], *p* = 0.049). Accordingly, for total T ≤ 6.2 nmol/L, the OR and 95% CI of having pathologic results at MRI for each unit decrease in total T was 2.15 [1.17;3.94], *p* = 0.013, whereas for total T > 6.2 nmol/L, it was 1.00 [0.73;1.37], *p* = 0.989. At total T 5.2 nmol/L, a significant increase of the estimated risk was observed for values below the threshold (*B* = 0.15 [0.02;0.27], *p* = 0.018 for each 1 nmol/L decrease in total T) but not above (*B* = 0.03 [− 0.02;0.09], *p* = 0.246). However, at total T 5.2 nmol/L, the change in the slope of the relationship above and below the threshold was not significant (*B* = 0.11 [− 0.04; 0.26], *p* = 0.139). Figure 2b, c shows the relationships of LH and FSH with the estimated probability of pathologic MRI. The background prevalence of abnormal MRI is exceeded for LH = 2.6 U/L and FSH = 3.8 U/L. However, the LOWESS curve did not clearly suggest a threshold effect and the regression with spline function confirmed the lack of a clear point above and below which the curves show different slopes. Figure 2d reports the LOWESS curve for PSA showing at 3.0 ng/mL the point, where the background prevalence of pathologic MRI is reached. However, the regression modeling with linear spline functions did not identify a threshold effect at that point. Conversely, at PSA = 0.77 ng/mL, a significant relationship with the risk of pathologic MRI for values below (*B* = 0.49 [0.04;0.93], *p* = 0.033) but not above this value (*B* = 0.02 [− 0.10;0.13], *p* = 0.740) was found, with a difference in the slope, which approached the statistical significance (*B* = 0.47 [− 0.04;0.97], *p* = 0.069). The OR of having pathologic MRI for each unit decrease in PSA below 0.77 ng/mL was 7.69 [1.08;54.88], *p* = 0.042, whereas above PSA 0.77 ng/mL the OR was 1.12 [0.63;1.98], *p* = 0.708.

**Fig. 3:**
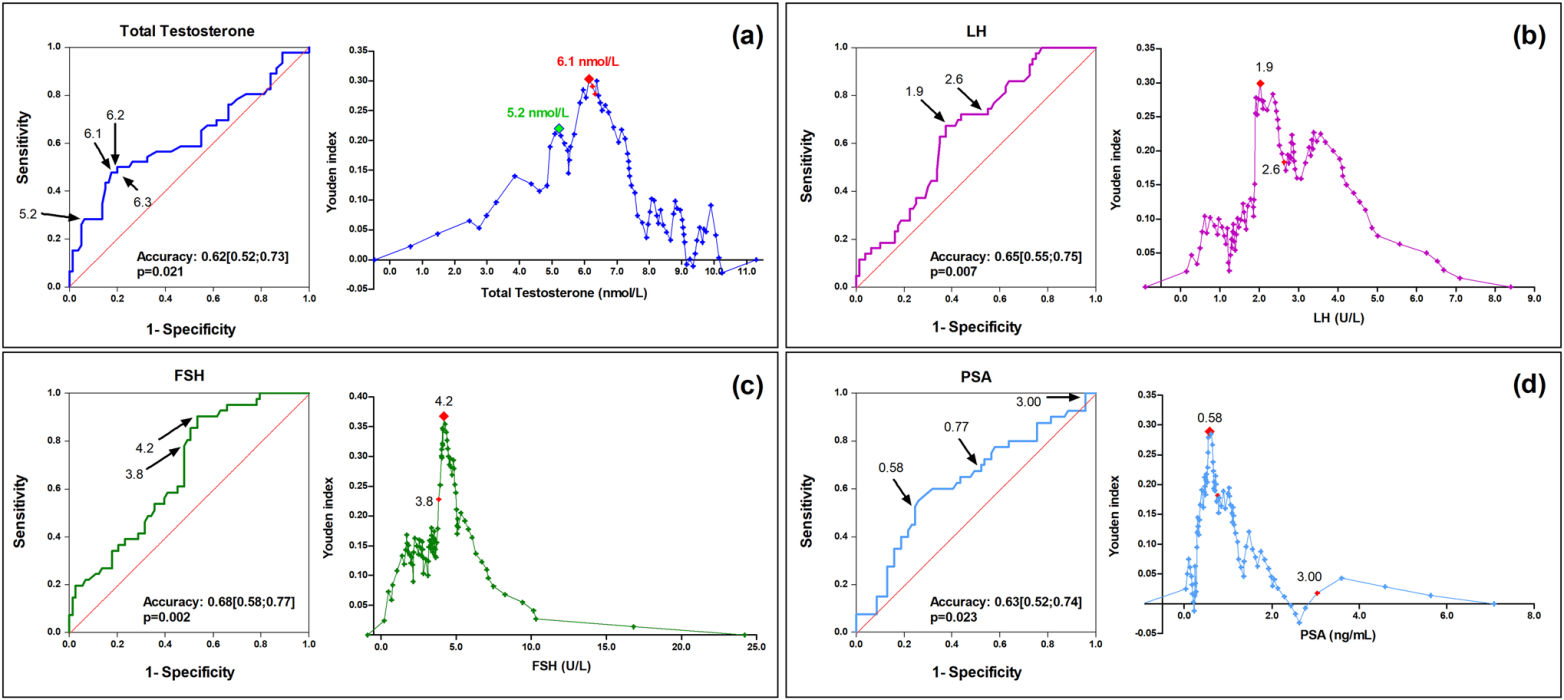
Receiver operating characteristics (ROC) analyses for the accuracy of total testosterone (**a**), LH (**b**), FSH (**c**), PSA (**d**) in discriminating men with normal or pathologic findings on hypothalamus–pituitary MRI. For each panel, the left side reports the ROC curve with arrows indicating the values of the serum parameter that emerged as relevant from the LOWESS or the ROC analyses or that correspond to the background prevalence of pathologic MRI in the UNIFI cohort. The right side of the panel shows the Youden index for each value of the serum parameter reported on the *x*-axis. *MRI* magnetic resonance imaging, *LH* Luteinizing hormone, *FSH* Follicles stimulating hormone, *PSA* Prostatic specific antigen, *LOWESS* locally weighted scatterplot smoothing

To evaluate the performance of possible cutoff values in discriminating men with secondary hypogonadism with or without pathologic findings at MRI, ROC analyses were carried out. Figure 3 reports these analyses for total T, LH, FSH and PSA. The accuracy of these values in predicting pathologic MRI was similar, their average ranging 0.62–0.68, all statistically significant (Fig. 3). The calculation of the Youden index allowed detecting, for each hormone and biochemical parameter, the value with the best performance that corresponded to total T 6.1 nmol/L, LH 1.9 U/L, FSH 4.2 U/L and PSA 0.58 ng/mL. Table 3 reports the sensitivity and specificity of these values together with those detected as the best threshold values at the LOWESS analyses and that corresponding to the background prevalence of pathologic MRI. Concerning total T 5.2 nmol/L, it corresponded to sensitivity 28.3% and specificity 93.8%.

**Table 3:** Sensitivity and specificity for the values with the best Youden index, for the threshold values identified at LOWESS and for the value correspondent to the background prevalence of pathologic MRI for total testosterone, LH, FSH and PSA

	Best Youden Index			Threshold value at LOWESS			Value correspondent to the background prevalence of pathologic MRI		
	Value	Sensitivity (%)	Specificity (%)	Value	Sensitivity (%)	Specificity (%)	Value	Sensitivity (%)	Specificity (%)
Total testosterone (nmol/L)	6.1	47.8	82.5	6.2	47.8	81.3	6.3	47.8	80.0
LH (U/L)	1.9	67.4	62.5	–	–	–	2.6	72.1	46.3
FSH (U/L)	4.2	90.2	46.6	–	–	–	3.8	78.7	52.1
PSA (ng/mL)	0.58	57.5	71.0	0.77	67.5	47.8	3.00	100.0	4.4

*LOWESS* Locally weighted scatterplot smoothing, *MRI* Magnetic resonance imaging, *LH* Luteinizing hormone, *FSH* Follicle stimulating hormone, *PSA* Prostatic specific antigen

### Bologna Maggiore Hospital cohort

To validate the values emerged as the most performant in the UNIFI cohort, we considered an independent cohort including a consecutive series of 50 men ascertained with secondary hypogonadism that underwent a hypothalamus–pituitary MRI. Their characteristics are reported in Table 4 and the specific findings of the positive MRIs are reported in the “Materials and methods” section.

**Table 4:** Characteristics of patients with secondary hypogonadism elected for hypothalamic–pituitary MRI at the Bologna Maggiore Hospital

	Bologna Maggiore Hospital cohort (*n* = 50)
Age (years)	50.9 ± 11.3
Current smoker (%)	18.0
Alcohol intake (> 4 drink/day) (%)	0.0
Testis volume (mL)	17.8 ± 4.2
Waist circumference (cm)	102.7 ± 13.4
Waist circumference > 102 cm (%)	52.9
BMI (kg/m^2^)	28.7 ± 5.3
Obesity (BMI > 30 kg/m^2^) (%)	35.3
Hypertension (%)	73.3%
Luteinizing hormone (U/L)*	1.9 [0.98–2.05]
Follicle stimulating hormone (U/L)*	2.8 [1.2–3.7]
Thyroid stimulating hormone (mU/L)*	1.4 [1.1–2.2]
Prolactin (mU/L)*	214.1 [107.5–355.0]
Overt hyperprolactinemia (PRL > 735 mU/L) (%)	12.5
Antipsychotics (%)	0.0
Prokinetics (%)	0.0
SSRI (%)	2.0
Total testosterone (nmol/L)	6.9 ± 2.4
Sex hormone binding globulin (nmol/L)*	24.0 [19.9–34.7]
Calculated free testosterone (pmol/L)	160.3 ± 57.1
Hemoglobin (g/dL)	14.8 ± 1.1
Prostate specific antigen (ng/mL)*	0.72 [0.32–1.06]
Total cholesterol (mg/dL)	207.9 ± 33.0
HDL-cholesterol (mg/dL)	44.5 ± 11.7
Triglycerides (mg/dL)*	139.0 [111.0–209.0]
Fasting glucose (mg/dL)	103.4 ± 35.4
Diabetes mellitus (%)	14.0
Severe erectile dysfunction (%)	57.1
Hypoactive sexual desire (%)	42.9
Impaired morning erection (%)	88.5
Perceived reduced ejaculate volume (%)	45.5

*MRI* magnetic resonance imaging, *BMI* body mass index, *PRL* prolactin, *SSRI* selective serotonin reuptake inhibitor, *HDL* high-density lipoprotein

The application of the aforementioned threshold values to this cohort, confirmed that total T 6.1 nmol/L and LH 1.9 U/L discriminates subjects with and without pathological findings at MRI (OR = 5.49 [1.32;22.85], *p* = 0.019 and OR = 3.31 [1.02;10.72], *p* = 0.046 for total T and LH, respectively). Conversely, neither FSH nor PSA were confirmed as discriminatory values in this validation cohort (OR = 2.19 [0.45;10.53], *p* = 0.329 and 0.70 [0.16;3.10], *p* = 0.638 for FSH ≤ 4.2 UL/ and PSA ≤ 0.58 ng/mL, respectively).

Interestingly, when evaluating subjects with total T below 6.1 nmol/L and LH above 1.9 U/L, the risk of pathological findings at MRI was significantly higher than in men with total T > 6.1 nmol/L and LH > 1.9 U/L (OR = 11.67 [1.11;122.38], *p* = 0.040) and not significantly different from patients with total T ≤ 6.1 nmol/L and LH ≤ 1.9 U/L (OR = 1.43 [0.10;20.44], *p* = 0.793). Similarly, men with total T above 6.1 nmol/L and LH below 1.9 U/L had significantly higher risk as compared with men with total T > 6.1 nmol/L and LH > 1.9 U/L (OR = 4.67 [1.11;19.65], *p* = 0.036) but not different from subjects with both low total T and LH (OR = 0.57 [0.09;3.83], *p* = 0.564).

### Sensitivity analysis

Table 5 shows the risk of pathological MRI among men from the UNIFI cohort with conditions known to induce functional secondary hypogonadism as well as the probability of abnormal MRI findings associated with low total T or LH in these subjects. Interestingly, even in men with metabolic conditions, the risk of having hypothalamic–pituitary abnormalities was around 40%. By applying the threshold of total T ≤ 6.1 or LH ≤ 1.9 U/L this risk was substantially increased up to 60% as found in diabetic men.

**Table 5:** Probability of pathological findings at MRI among men with metabolic conditions possibly leading to secondary hypogonadism

Possible functional causes	Probability of pathological MRI		
	Whole group (%)	Men with total T ≤ 6.1 nmol/L (%)	Men with LH ≤ 1.9 U/L (%)
Diabetes mellitus	40.0	42.9	100.0
Hypertension	37.0	62.1	47.2
Dyslipidemia	43.6	70.0	47.2
BMI ≥ 30 kg/m^2^	41.3	46.2	54.5
Waist circumference > 102 cm	43.5	53.3	53.3

*MRI* magnetic resonance imaging; *T* testosterone; *LH* luteinizing hormone

After excluding from the UNIFI cohort 11 men with overt hyperprolactinemia, the sensitivity and specificity for pathologic MRI detection were, respectively, 28.6% and 94.8% for total T ≤ 5.2 nmol/L, 45.7% and 83.1% for total T ≤ 6.1 nmol/L, and 57.6% and 66.2% for LH ≤ 1.9 U/L.

The predicting performance of total T ≤ 6.1 nmol/L and LH ≤ 1.9 U/L in the Bologna Maggiore Hospital cohort, after the exclusion of 6 overtly hyperprolactinemic men, were quantified, respectively, by OR = 8.00 [1.28;50.04], *p* = 0.026 and OR = 3.67 [0.70;19.12], *p* = 0.123.

## Discussion

This study shows that, in a population of men with low T due to inappropriate hypothalamic–pituitary stimulation, the threshold of total T 5.2 nmol/L, suggested by the ES [7], is helpful in discriminating subjects with pathologic MRI findings. Indeed, our data confirm that, based on excellent specificity, total T below 5.2 nmol/L may definitively rise the suspicion of structural hypothalamic–pituitary alterations; however, in this cohort, it is associated with low sensitivity, thus leaving undiagnosed a significant proportion of men with structural hypothalamic–pituitary abnormalities. According to the present results, total T 6.1 nmol/L is a more suitable threshold. This value was identified by a series of robust statistical analyses aimed at selecting, with different approaches, a series of possible proper values. These were close each other and total T 6.1 nmol/L was eventually identified based on better operating characteristics, as denoted by the Youden index, which was the highest in the whole total T range. To our knowledge, this is the first study to identify formally, with a similar approach, a threshold for LH (i.e., 1.9 U/L), which suggests pathologic MRI findings. The cutoff values for LH and total T emerged by these results provide enough confidence, because they find confirmation in an independent validation sample (Bologna Maggiore Hospital cohort). FSH and PSA have been tested as further possible parameters. However, despite in the training population (UNIFI cohort) they showed threshold values with interesting performance measures, these were not confirmed in the validation sample. This prevents to support their use in clinical practice for this specific purpose, although they may deserve further assessment in future studies.

The role of total T as a hallmark of possible pathologic MRI findings in secondary hypogonadal patients has been previously considered by a limited number of studies [8, 14–19] and, among these, only one have recognized a threshold for total T [8]. This latter study has the value to have attempted a formal approach for establishing a threshold, by evaluating the difference in the frequency of abnormal computed tomography (CT) or MRI findings among total T quintiles. On the other hand, a drawback is the arbitrary definition of secondary hypogonadism according to total T < 8 nmol/L and LH < 13 U/L, which is likely to include hypogonadal subjects with primary testicular impairment. This may have biased the results obtained and led to reduce the threshold value for identifying abnormal hypothalamic–pituitary findings. Accordingly, a study conducted on 281 secondary hypogonadal men—defined by hormone criteria closer to ours (total T < 10 nmol/L and LH < 10 U/L)—who underwent a pituitary MRI showed that more than half of those carrying hypothalamic–pituitary lesions had total T above 5.2 nmol/L [15]. A study comparing secondary hypogonadal men with hypothalamus–pituitary structural abnormalities with late onset hypogonadal men [19] confirmed a worsening in sensitivity as total T levels get lower and, interestingly, reported at total T < 6.8 nmol/L operating characteristics comparable to those found in the present study at 6.1 nmol/L. A study on 75 secondary hypogonadal men with total T < 10 nmol/L and LH < 10 U/L [14] did not find significant differences in the prevalence of pathologic CT or MRI findings throughout different T quartiles, although a step of frequency was present at total T around 6.2–7.4 nmol/L, which compares well with our results. The non-negligible frequency of radiologic abnormalities (around 17%) reported by this study even in men with total T ranging 7.4–10.0 nmol/L is also in line with the present results. In fact, it is interesting to note that, at variance with gonadotropins, the risk of pathologic MRI in our sample does not approach zero even for the highest total T values. This is tantamount to say that in secondary hypogonadal patients, underlying anatomical abnormalities could never be completely ruled out by considering only T. In this context, the identification of further biochemical markers may be useful. Gonadotropins appears as the most obvious parameters to be considered. Because the difference in gonadotropin levels in subjects with or without hypothalamus–pituitary abnormalities did not achieve statistical significance in previous studies [8, 15–17], they did not emerge as possible discriminating parameters before. A recent study [20] performed in 141 mildly hyperprolactinemic men with sexual, fertility or fatigue complaints, showed that, besides lower total T and higher prolactin, subjects with pituitary abnormalities had significantly lower LH levels. However, LH was eventually proposed as a third-line evaluation (after prolactin and total T) based on the putative lower likelihood of being measured in clinical practice for the management of such population [20]. A LH threshold of 5 U/L was arbitrarily set to capture pathologic MRI findings in men with low prolactin-to-T ratio, although the addition of LH to prolactin and total T was not associated with greater accuracy of the predicting model for pituitary abnormalities [20]. Different from this study, the present one—performed on secondary hypogonadal men rather than mild hyperprolactinemic ones—used a formal approach to define LH 1.9 U/L as the best threshold value to detect hypothalamic–pituitary abnormalities. It is noteworthy that, when applying LH ≤ 1.9 U/L to detect structural hypothalamic–pituitary abnormalities in men with total T > 6.1 nmol/L, it appeared as a significant predictor in the validation sample (Bologna Maggiore Hospital cohort), associated with more than threefold higher risk than men with both hormones above the cutoff and with comparable risk of those with both hormones low. This suggests that, in secondary hypogonadal men, low LH, independently of total T, may rise the suspicion of organic conditions affecting the hypothalamus and/or the pituitary, in contrast with the results of the aforementioned study on hyperprolactinemic men [20], which outpaced LH as a second line and redundant marker. However, it should be recognized that, also from the present results, low total T emerges as the strongest predictor. Indeed, when applying total T ≤ 6.1 nmol/L to secondary hypogonadal men with LH > 1.9 U/L, the risk of abnormal MRI reports in the validation sample was more than tenfold higher than in men with total T and LH above the threshold.

Similar to LH, FSH has been tested in the exploratory sample (UNIFI cohort) because of significantly different values between men with or without abnormal MRI. Further analyses showed that FSH has a good accuracy in detecting pathologic MRIs. FSH ≤ 4.2 U/L is characterized by the best Youden index, however, derived by the combination of high sensitivity and low specificity, the latter carrying the risk of high false positive rate and unnecessary MRIs required. Accordingly, when the value is plotted in the LOWESS curves, it corresponds to a risk of pathologic MRI below the background prevalence of the whole population. The attempt to test the predictive value of FSH ≤ 4.2 U/L in the validation cohort did not support its use as a predictor. However, if confirmed in larger populations, the operating characteristics of FSH thresholds counterbalance those of total T thus suggesting that their combined use may improve the predicting value.

PSA also emerged as a possible discriminatory value by the comparison of the groups with or without structural hypothalamus–pituitary abnormalities. Although the production of this protein is outside the HPT axis, and thus, its low values are not immediately related to hypothalamus–pituitary anatomical damage, the emergence of this values, among those candidate to further testing in the exploratory sample is far to be casual. In fact, the prostate is deeply affected by T in either its trophic or biochemical activities [21], including PSA secretion. Accordingly, we previously reported that PSA values are in an S-shaped relationship with total T, and PSA < 0.65 ng/mL could be regarded as a truthful marker of hypogonadism [22]. Since PSA codifying gene has two androgen responsive elements [23], its circulating levels reflect T bioactivity and may be less affected by the day-to-day fluctuations that notoriously T has [24]. In this view, PSA may have represented an interesting predictor of MRI findings. Indeed, its accuracy in the UNIFI cohort is comparable with the HPT hormones and the operating characteristics of the values identified by the LOWESS and the ROC curves analysis (PSA ≤ 0.77 and ≤ 0.58 ng/mL, respectively) possibly useful. However, the attempt to validate these thresholds in the Bologna Maggiore Hospital cohort did not confirm their predicting role and does not allow, at present, supporting PSA as a possible predictor of MRI findings in secondary hypogonadal men.

This study has some limitations. Firstly, MRIs were not required to every secondary hypogonadal patient but the decision of performing an MRI entirely relied upon the physician decision. When comparing those for whom MRI was or was not required, it appeared that endocrinologists at our center are more prone to test further younger non-diabetic men, with decreased sexual desire as the predominant sexual symptom and those with overt hyperprolactinemia, lower total T and gonadotropin levels. This may have biased the present results to identify lower thresholds that could leave undiagnosed a fraction of patients. However, all the studies so far published on this topic have the same drawback, including the one from Citron et al. [8] that inspired the suggestion from the ES guidelines. Accordingly, the use of a slightly higher threshold for total T emerged from the present results may be even more justified. A further limitation is the relatively small sample size; however, it is representative of the 15-year clinical practice of a third level Andrology University center and it compares well with similar casuistries [8, 14, 16, 17].

The study has also some strengths. The robust statistical approach, which used different methods to define a set of possible threshold values and the independent cohort to validate the findings obtained in the exploratory cohort represent the major strengths. Previous studies used rougher methods (i.e., percentiles) that are more suitable to indicate a range of values around the limit of the percentile rather than a precise number on the whole range of the possible values. In addition, we did not operate a selection on the abnormalities reported on the MRI reports and a larger set of abnormalities (not only micro- and macro-adenomas) is included among the pathologic MRI. This extends the applicability and usefulness of our results, because the threshold values here proposed aim at identifying all the anatomical abnormalities that could participate in determining an inappropriate hypothalamus–pituitary response to T decline. This is important in the context of the dichotomy organic vs. functional hypogonadism, because it may help identifying men that, although having concomitant conditions favoring functional impairment of HPT, could be not entirely able to improve secondary hypogonadism upon correction of the underlying condition. A further strength is that all our hypothalamus–pituitary imaging was performed by MRI, rather than CT or other imaging, which has been demonstrated to be the most sensitive in revealing focal abnormalities within normal pituitary gland [25, 26].

## Conclusions

In subjects diagnosed with secondary hypogonadism and consulting for sexual dysfunction, hormonal parameters can help in recognizing men who deserve performing hypothalamus–pituitary MRI. In particular, the present results support total T ≤ 6.1 nmol/L as a valid threshold value to adequately recognize patients with hypothalamic–pituitary structural alterations. This is close to total T 5.2 nmol/L suggested by the ES but it allows a moderate gain in sensitivity, with a relatively preserved specificity, thus limiting the rate of patients erroneously left with undiagnosed hypothalamus–pituitary abnormalities. Posing more value on limiting false negative rate is important, because organic hypothalamus–pituitary conditions require a periodic follow-up to evaluate their potential development and consequences on pituitary function. Moreover, the exclusion of organic pathogenesis of secondary hypogonadism is necessary to definitively conclude for a functional condition and to consequently consider lifestyle modifications or comorbidity treatment optimization as valid therapeutic options. The present study, for the first time, introduce LH as a useful predictor, being 1.9 U/L a proper threshold able to help in the decision-making about the diagnostic workup in secondary hypogonadal men. Interestingly, either total T ≤ 6.1 nmol/L or LH ≤ 1.9 U/L can be used independently of each other to decide on MRI testing. Larger studies are required to confirm our findings and to test FSH and PSA as further predictors based on promising operating characteristics found by the present study.
